# A nomogram based on radiological features of MRI for predicting the risk of severe pain in patients with osteoarthritis of the knee

**DOI:** 10.3389/fsurg.2023.1030164

**Published:** 2023-02-08

**Authors:** Zhuce Shao, Zhipeng Liang, Peng Hu, Shuxiong Bi

**Affiliations:** Department of Bone and Joint, Third Hospital of Shanxi Medical University, Shanxi Bethune Hospital, Shanxi Academy of Medical Sciences, Tongji Shanxi Hospital, Taiyuan, China

**Keywords:** KOA, K–L, nomogram, forecast, pain, TKA

## Abstract

**Methods:**

This study aimed to develop and validate a nomogram for predicting the risk of severe pain in patients with knee osteoarthritis. A total of 150 patients with knee osteoarthritis were enrolled from our hospital, and nomogram was established through a validation cohort (*n* = 150). An internal validation cohort (*n* = 64) was applied to validate the model.

**Results:**

Eight important variables were identified using the Least absolute shrinkage and selection operator (LASSO) and then a nomogram was developed by Logistics regression analysis. The accuracy of the nomogram was determined based on the C-index, calibration plots, and Receiver Operating Characteristic (ROC) curves. Decision curves were plotted to assess the benefits of the nomogram in clinical decision-making. Several variables were employed to predict severe pain in knee osteoarthritis, including sex, age, height, body mass index (BMI), affected side, Kellgren—Lawrance (K–L) degree, pain during walking, pain going up and down stairs, pain sitting or lying down, pain standing, pain sleeping, cartilage score, Bone marrow lesion (BML) score, synovitis score, patellofemoral synovitis, bone wear score, patellofemoral bone wear, and bone wear scores. The LASSO regression results showed that BMI, affected side, duration of knee osteoarthritis, meniscus score, meniscus displacement, BML score, synovitis score, and bone wear score were the most significant risk factors predicting severe pain.

**Conclusions:**

Based on the eight factors, a nomogram model was developed. The C-index of the model was 0.892 (95% CI: 0.839–0.945), and the C-index of the internal validation was 0.822 (95% CI: 0.722–0.922). Analysis of the ROC curve of the nomogram showed that the nomogram had high accuracy in predicting the occurrence of severe pain [Area Under the Curve (AUC) = 0.892] in patients with knee osteoarthritis (KOA). The calibration curves showed that the prediction model was highly consistent. Decision curve analysis (DCA) showed a higher net benefit for decision-making using the developed nomogram, especially in the >0.1 and <0.86 threshold probability intervals. These findings demonstrate that the nomogram can predict patient prognosis and guide personalized treatment.

## Introduction

Osteoarthritis (OA) is one of the most important causes of pain and disability in the elderly in recent years (1). It mainly affect the mobility and other functions of the knee joint (2), and is characterized by degenerative changes in the joints that usually cause pain, stiffness and reduced joint function (3–6). The increase in aging population has increased the number of patients with osteoarthritis of the knee, with osteoarthritis of the knee (KOA) affecting close to 20% of the world population (7). This inevitably reduces the quality of life of patients and imposes a substantial medical burden on the society (8).

According to clinical guidelines, the focus of osteoarthritis of the knee treatments is to relieve pain (9). Severe pain level in patients with knee osteoarthritis is the most important reason why patients seek medical care (10–13). The available treatments include conservative treatment, total knee arthroplasty, or open wedge high tibial osteotomy (OWHTO) (14–21). As with many chronic pain disorders, the mechanisms of pain in knee OA are complex, multifactorial mixed, and incompletely understood (22, 23). Many studies have shown that the radiological presentation of disease severity does not agree with patient pain severity and disability (24–26), suggesting that many other factors may also or more meaningfully contribute to changes in joint pain in patients with OA (27–29).

There is a need to investigate the pathology of knee osteoarthritis in terms of pain development and the factors influencing it. It was generally believed that the onset of KOA pain was due to mechanical loading or aging injury (30), but recent studies have found that these causes cannot fully explain the degree of pain (31), given the significant inconsistencies between imaging findings and pain levels (32).

Identification of factors associated with severe pain may promote timely detection and early intervention. This will reduce the pain and burden on the patient. Therefore, we aimed to develop a model for predicting the risk of severe pain.

## Materials and methods

### Patient selection

A total of 150 KOA patients treated at the Third Hospital of Shanxi Medical University from 2018 to 2020 were retrospectively enrolled in this study. An additional 64 internal validation patients were collected based on the following inclusion criteria.

**Inclusion criteria** (1). Patients who underwent TKA at the Department of Orthopedics, Third Hospital of Shanxi Medical University; (2). Patients with complete medical records; (3). Underwent first unilateral total knee arthroplasty.

**Exclusion criteria** (1). Patients with severe heart, liver, lung, and kidney organ dysfunction; (2). Patients with incomplete data; (3). Patients who were unable to cooperate, unwilling to participate, or lost to follow-up.

The study of this columnar graph prediction model was reviewed and approved by the Ethics Committee of the Third Affiliated Hospital of Shanxi Medical University. This study was conducted following the Declaration of Helsinki.

### Data collection

General information about the patient, clinical presentation, and, most importantly, some critical information about the patient′s MRI were collected and counted.

## Imaging assessment

### x-Ray assessment

The K–L grading scale which is extensively used in KOA studies as adopted to assess x-Ray results. We have standardized the method of x-ray examination of the patient to the frontal and lateral knee position, and the patient′s position during the examination to the standing position. The severity of bone wear in the knee joint in KOA was scored on x-ray as follows, Grade 0: Normal; Grade 1: Suspicious narrowing of the joint space with possible bone piles; Grade 2: Mild narrowing of the joint space with significant small bone piles; Grade 3. Significant narrowing of the joint space, as well as a moderate amount of bone stumps, mild sclerosis of the subchondral bone, and possible knee deformity; Grade 4: Severe narrowing of the joint space, extensive bone stump formation, as well as significant sclerosis of the subchondral bone and significant knee deformity.

### MRI evaluation

Patients received a Philips 3.0 T knee MRI scan of the affected knee. Image sequences included: (1) T1-weighted 3D fat-suppressed gradient echo sequence with 90° flip angle, TR: minimum500 ms, maximun 700 ms, FOV: 170 × 170 mm, TE: 20 ms, matrix: 380 × 305. Layer thickness: 4.0 mm, 19 layers scanned. (2) T2-weighted 3D fat-suppressed fast spin-echo sequence, flip angle 90°, FOV: 170 × 170 mm, TR: 500 ms, TE: 20 ms, matrix 340 × 265, layer thickness 4.0 mm, 19 layers scanned. The MRI images of the knee were evaluated observationally using the StartWebclient software.

**Figure 1:**
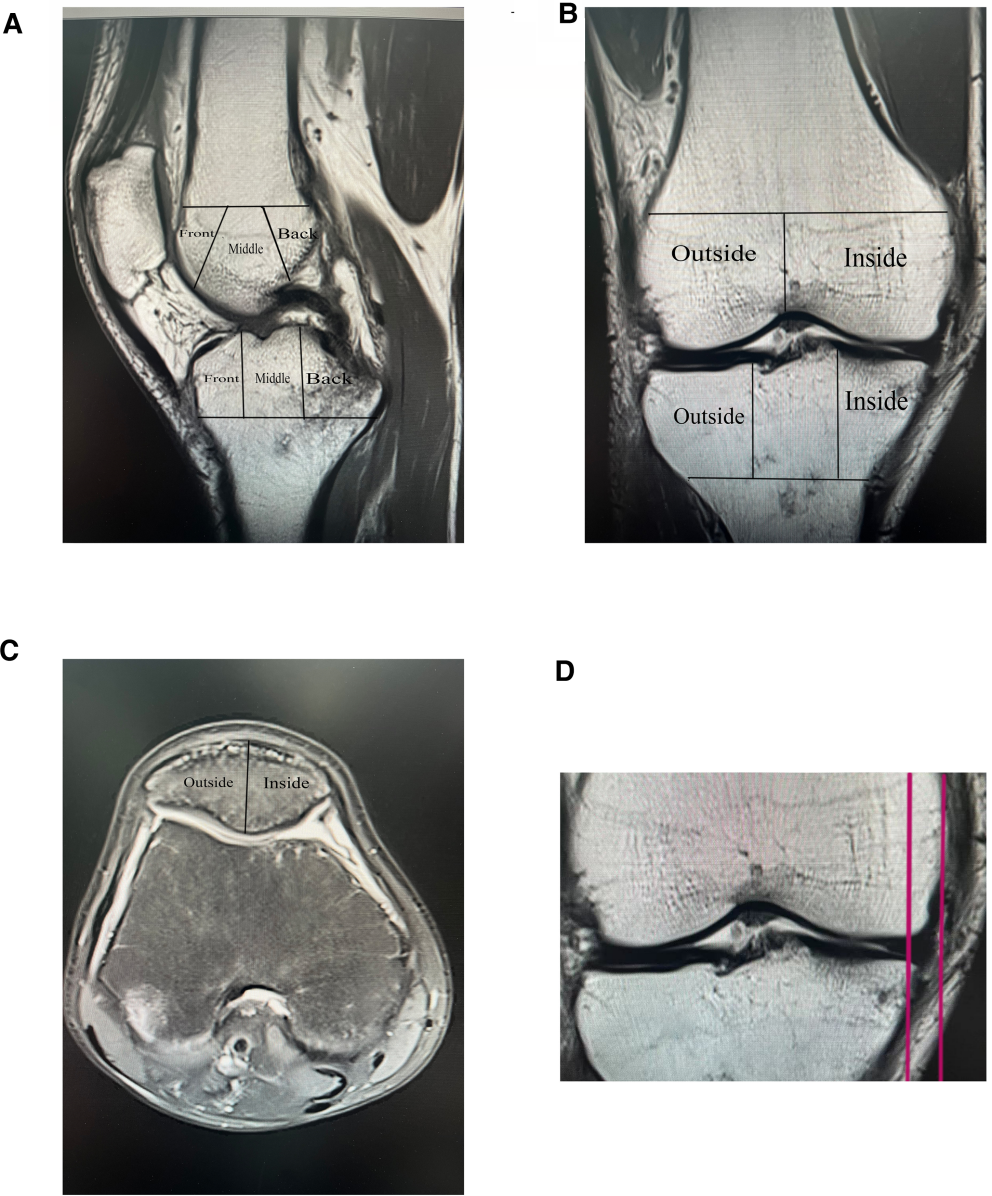
Imaging assessment, WORMS score and meniscus displacement map, (**A**, **B**, and **C**) Demonstrate the method of delineating regions for The Whole-Organ Magnetic Resonance Imaging Score (WORMS). (**D**) Shows an MRI coronal T1 image with an example of meniscus displacement, which in this illustration measures 6.1 mm.

In 1957, Kellgren and Lawrence (KL) first established and used the imaging classification scheme for OA, the K–L grading scoring system that was later used with our present study (33). Whole-Organ Magnetic Resonance Imaging Score (WORMS) is a knee scoring system for whole-organ assessment of the knee in knee osteoarthritis based on MRI (34). The Whole-Organ Magnetic Resonance Imaging Score (WORMS) divides the knee into 15 regions, and the BML score is measured in a T2 image with grade 0 normal, grade 1 less than 33% of the region, grade 2 accounting for 33%–66% of the altered area and grade 3 more significant than 66% of the modified part. The total score is the sum of the regional scores. The delineated areas, as represented in Figures 1A–C, also explain some of our approaches to the study.

Synovitis score rating is based on the maximum expansion of the synovial cavity, with grade 0 typical (physiological effusion), grade 1 less than 33% of the total potential increase, grade 2 accounting for 33%–66% of the maximum possible expansion, and grade 3 more significant than 66% of the maximum potential growth.

### Meniscus displacement

To record T1 image, measuring the distance between the plumb line of the largest section of the meniscus and the plumb line over the edge of the tibial plateau was recorded. Meniscus displacement was measured on T1 images in the coronal MRI position, with two vertical lines drawn in the image, one over the largest section of the medial or lateral meniscal volume and the other over the edge of the medial or lateral tibial plateau (excluding the osteophyte), and the distance between the two vertical lines was defined as a measure of Meniscus displacement, as shown in Figure 1D. For example, Figure 1D is an MRI coronal T1 image with a meniscal displacement measurement of 6.1 mm.

Bone marrow lesion (BML) was histologically defined as bone marrow necrosis, trabecular bone abnormalities, bone marrow fibrosis, edema, cellular infiltration, and vascular hyperplasia. Magnetic Resonance Imaging (MRI) presents as a T1-weighted image with low signal and a T2-weighted image with high signal short-time inversion recovery sequence (STIR) high signal images in a punctate, map-like, or diffuse irregular pattern. Bone marrow lesions (BML) are measured in T2 images on MRI and are graded as follows, grade 0: normal; grade 1:≤33% of the area; grade 2: 33%–66% of the area; grade 3:≥66% of the area.

Observations and diagnostic scores were assigned by two attending physicians who do not know each other, and if the results deviated significantly from the values, further assessments were made by a more senior and highly qualified chief physician with many years of experience.

Pain was expressed based on the McMaster Universities Osteoarthritis Index (WOMAC) pain score, using the Western Ontario and McMaster Universities Osteoarthritis Index (WOMAC), the degree of pain in the patient′s knee was determined (35, 36). The questionnaire is a valid, reliable, and highly sensitive indicator, and the pain component consists of five items (walking on a flat surface, walking up/down stairs at night, sitting/reclining, and standing upright), each with five levels (0 = none, 4 = severe) and a total score of 20. The entire score was divided into two parts, with a score greater than ten considered an extreme pain level.

The knee was divided into 15 observation areas according to the WORMS scale as shown in Figures 1A,B: The medial femoral condyle, lateral femoral condyle, medial tibial plateau and lateral tibial plateau are each divided into three regions: anterior, middle and posterior (the trochanter of the femur is considered to be part of the medial condyle), and the subspinous (S) region of the tibial intercondylar spine is an area without cartilage coverage. In Figure 1C, the patella was divided into medial and lateral. Figure 1D shows the MRI coronal T1 image with a meniscal displacement measurement of 6.1 mm.

The remaining variables, except BMI, were converted to binary variables. Patients’ outcome times were divided into two groups: the severe pain group and the non-severe pain group. The score for pain was determined by averaging the scores of two senior doctors in our department. The imaging assessment was conducted jointly by two experienced senior physicians from our imaging department.

### Statistical analysis

Statistical analysis was performed by SPSS 25.0 (IBM, Chicago, IL, USA) and R for windows (version 4.0.5, http://www.r-project.org/). Firstly, Lasso regression was used to select significant variables, logistics regression analysis was used to display risk factors, and a nomogram chart was constructed according to the RMS package′s univariate and multivariate analysis results. The accuracy of the nomogram was tested using the modeling cohort and the internal validation cohort through identification and calibration. Finally, C-index, calibration curve, and ROC curve were constructed to evaluate the accuracy of the prediction model. The DCA curve was also employed to assess the effectiveness of the nomogram in clinical applications.

## Results

### Patient characteristics

In the modeled population, 36.7% of the patients were male, and 63.3% were female. In addition, most patients (69.3%) had a BMI >24.0. Moreover, 35.3% (53) patients had severe pain. Other clinical data of patients are presented in Table 1.

**Table 1:** Patient demographic characteristics of the modeled population.

Characteristic	Training cohort (*n* = 150)/*n* (%)	Validation cohort (*n* = 64)/*n* (%)	*P* value
Age (%)			0.132
≤60	80 (53.3)	37(57.8)	
>60	70(46.7)	27 (42.2)	
Sex (%)			0.878
Male	55 (63.3)	43(67.2)	
Female	95 (36.7)	21 (32.8)	
BMI (%)			0.102
<18.5	3(2.0)	5 (7.8)	
18.5–24.0	43 (28.7)	7(10.9)	
>24	104 (69.3)	52 (81.2)	
Laterality (%)			0.144
Left	80 (53.3)	33 (51.6)	
Right	70 (46.7)	31 (48.4)	
Time (%)			0.428
≤12	94 (62.7)	46(71.9)	
>12	56 (37.3)	18 (28.1)	
K-Lgrade (%)			0.356
≤2	67 (44.7)	29 (45.3)	
>2	83 (55.3)	35 (54.7)	
Cartilage_score (%)			0.554
≤20	106 (70.7)	40(62.5)	
>20	44(29.3)	24 (37.5)	
BML (%)			0.533
0–15	107 (71.3)	41(64.1)	
>15	43(28.7)	23 (35.9)	
synovitis_score (%)			0.122
0,1	94 (62.7)	37(57.8)	
2,3	56 (37.3)	27 (42.2)	
Osteophyte_score (%)			0.105
No	24 (16.0)	11(17.2)	
Yes	126 (84.0)	53 (82.8)	
Bone_wear_score (%)			0.477
No	113 (75.3)	45(70.3)	
Yes	37 (24.7)	19 (29.7)	
Meniscus_score (%)			0.184
No	47 (31.3)	40(62.5)	
Yes	103 (68.7)	24 (37.5)	
Meniscus_displacement (%)			0.807
No	91 (26.4)	26(40.6)	
Yes	59(73.6)	38 (59.4)	
Ligament (%)			0.424
No	121 (80.7)	49(76.6)	
Yes	29 (19.3)	15 (23.4)	
Status (%)			0.773
Non severe pain	97 (64.7)	40(62.5)	
Severe pain	53 (35.3)	24 (37.5)	

### Feature selection

**Figure 2:**
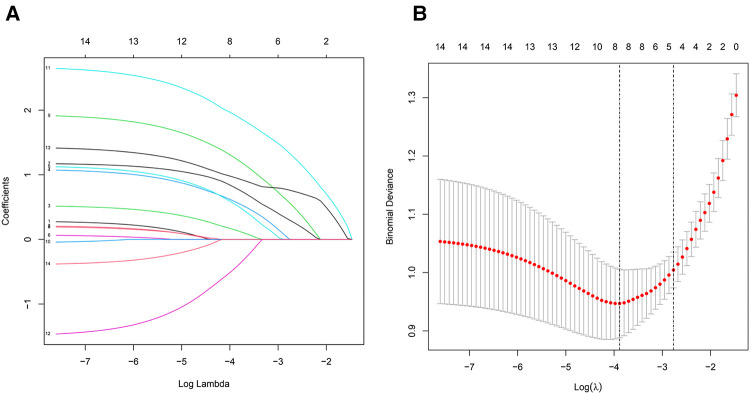
(**A**) All parameters were included in the LASSO analysis, and coefficient profiles were plotted for 14 features using the LASSO binary logistic regression model. (**B**) All parameters were included in the LASSO analysis. Binomial deviations were plotted using the LASSO binary logistic regression model, with eight parameters being statistically significant.

Among the 14 characteristics screened, 8 were considered potential predictors in 150 patients (∼2:1 ratio) and had non-zero coefficients in the LASSO regression model. These factors included BMI, affected side, duration of knee osteoarthritis, meniscus score, meniscus displacement, BML score, synovitis score, and bone wear score. The specific content is shown in (Figure 2).

### Logistics univariate and multivariate regression analysis of severe pain in patients with KOA

Logistic regression results showed that factors including BML score, synovitis score, bone wear score and meniscus displacement among others were more predictive of severe pain and were risk factors for severe pain in patients with KOA. The regression coefficients of 8 variables and other analysis results are shown in Tables 2, 3.

**Table 2:** Logistics regression univariate analysis.

	Univariate Analysis			
Variable	*β*	OR	95%CI	*P* value
BMI	0.515	1.674	0.876–3.5041	0.152
Laterality	0.501	1.650	0.843–3.260	0.145
Time	0.766	2.151	1.081–4.312	0.030
Meniscus_score	0.507	1.661	0.796–3.612	0.186
BML	1.66	5.257	2.4920–11.464	0.000
Synovitis_score	1.141	3.133	1.566–6.375	0.001
Bone_wearscore	2.393	10.951	4.736–27.496	0.000
Meniscus_displacement	2.065	7.884	3.778–17.189	0.000

**Table 3:** Logistics regression multifactor analysis.

	Multivariate Analysis			
Variable	*β*	OR	95%CI	*P* value
BMI	0.441	1.554	0.604–4.324	0.374
Laterality	1.028	2.797	1.117–7.426	0.032
Time	1.14	3.128	1.165–8.859	0.026
Meniscus_score	−1.289	0.276	0.081–0.853	0.030
BML	1.318	3.734	1.245–11.650	0.020
Synovitis_score	1.925	6.856	2.493–21.255	0.000
Bone_wearscore	2.529	12.541	4.011–44.548	0.000
Meniscus_displacement	1.278	3.591	1.265–10.514	0.017

*β* is the regression coefficient.

CI, confidence interval; OR, odds ratio.

### Establishment of a nomogram for predicting severe pain in KOA patients

The LASSO regression analysis results revealed that the BMI was a significant variable. Although the *P*-value for BMI was more than 0.05 in our logistic univariate and multivariate regressions, we concluded that the BMI was a clinically significant risk factor for pain in osteoarthritis of the knee. Therefore, we included the BMI in the nomogram.

**Figure 3:**
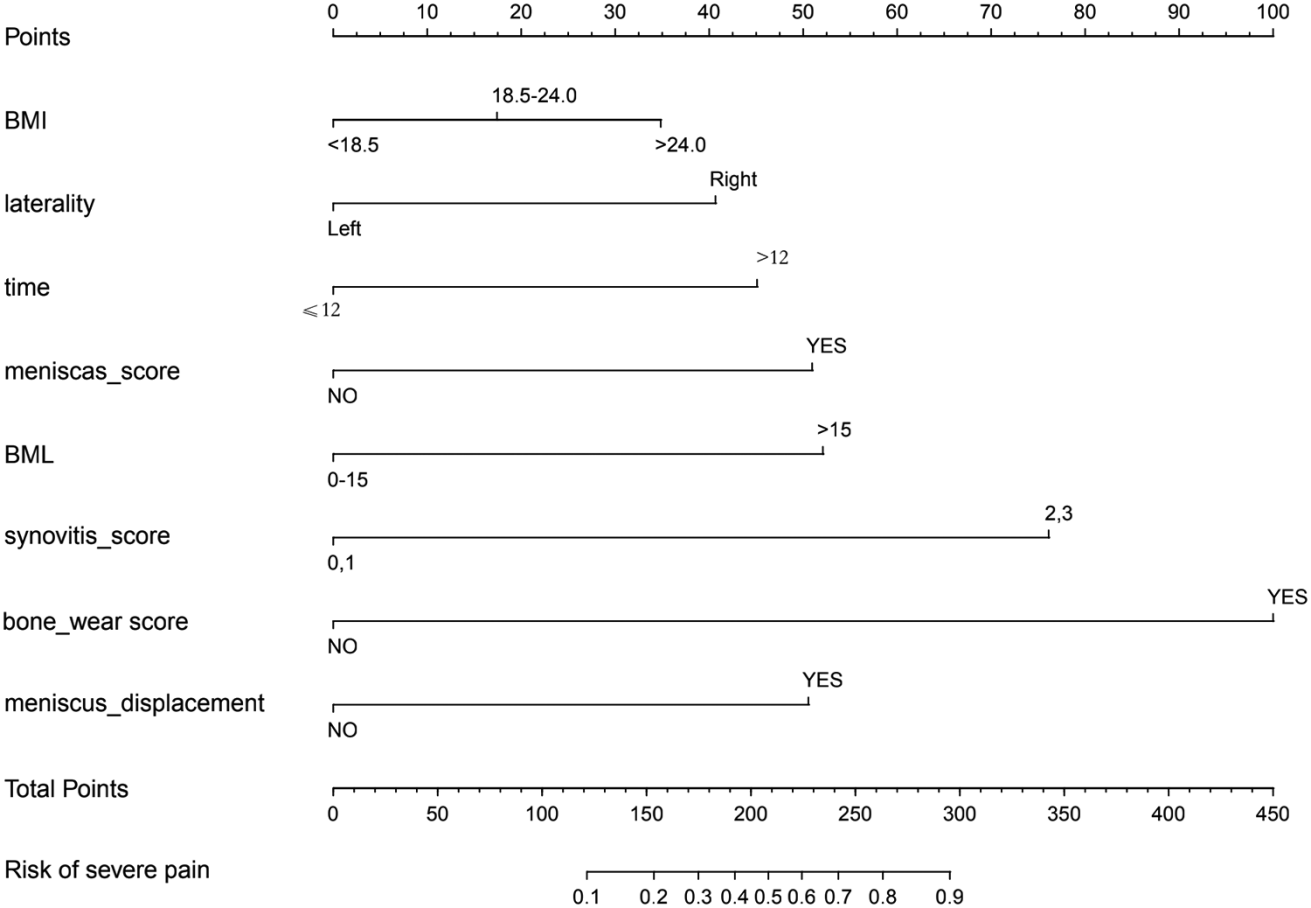
Nomogram drawn with 8 variables: BMI, affected side, duration of knee osteoarthritis, meniscal score, meniscal displacement, BML score, synovitis score, and bone wear score.

A nomogram for predicting the risk of severe pain in patients with KOA was constructed using eight variables: BMI, affected side, duration of knee osteoarthritis, meniscal score, meniscal displacement, BML score, synovitis score, and bone wear score. See Figure 3 for details of the nomogram (Figure 3).

### The calibration chart for validating the prediction accuracy of the nomogram

**Figure 4:**
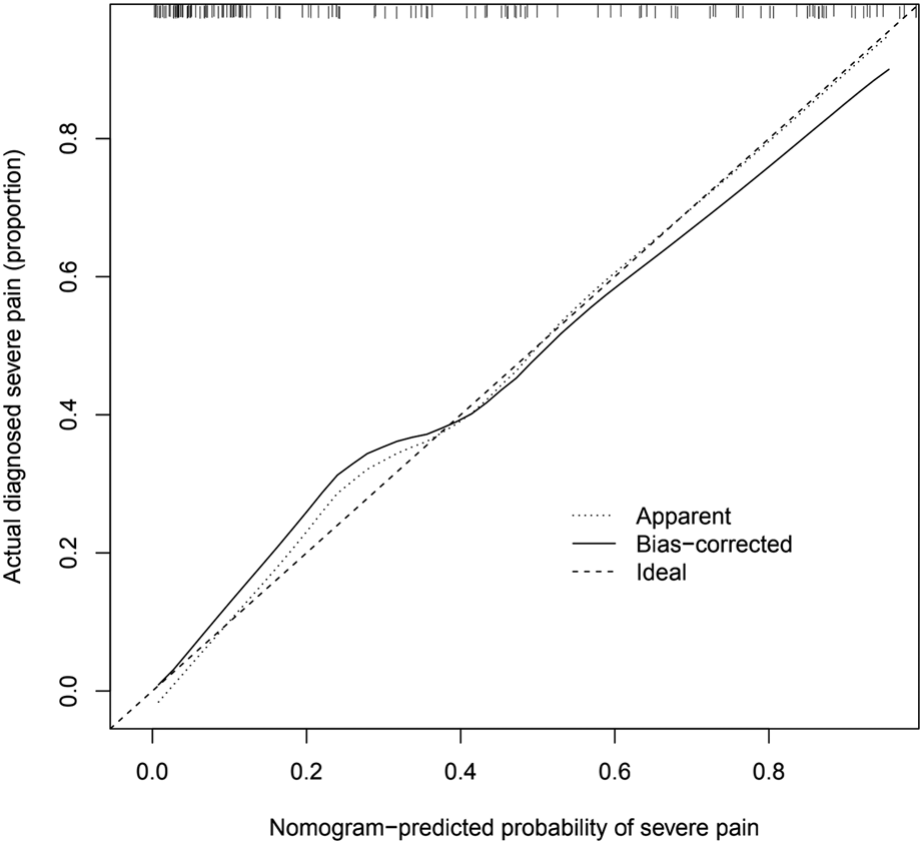
Calibration diagram of nomogram of modeling group.

Analysis of the calibration curve of the nomogram showed good agreement in this cohort. The C-index of the cohort prediction nomogram was 0.892 (95% CI:0.839–0.945), and for the internal validation it was 0.822 (95%: CI 0.722–0.922), indicating good discriminatory performance of the model. The nomogram showed good predictive power in predicting the occurrence of severe pain in KOA patients. The specific data of patients are shown in (Figure 4).

### ROC curve verifies the prediction accuracy of nomogram

**Figure 5:**
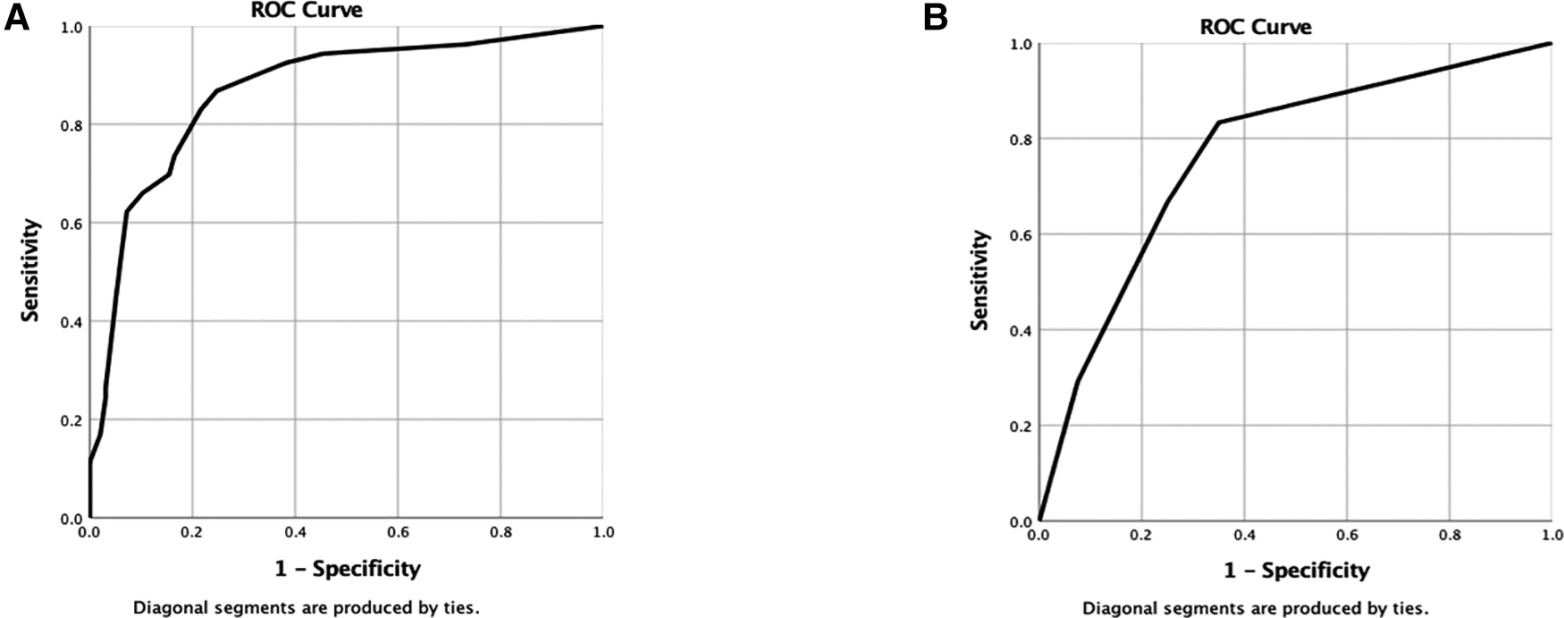
(**A,B**) Are the ROC curves of nomogram of modeling group and verification group respectively.

The area under the ROC curve (AUC) analysis was used to assess the accuracy of the model. The higher the AUC value, the better the model accuracy. The results showed that the model for predicting the risk rate of severe pain in KOA patients had better accuracy, as seen in the modeling cohort (AUC = 0.892). The final result are present in Figure 5A. In the internal validation cohort, the AUC value was 0.822. Other specific data of patients are shown in Figure 5B. Detailed results are presented in (Figure 5).

### Evaluating the clinical application of nomogram through DCA

**Figure 6:**
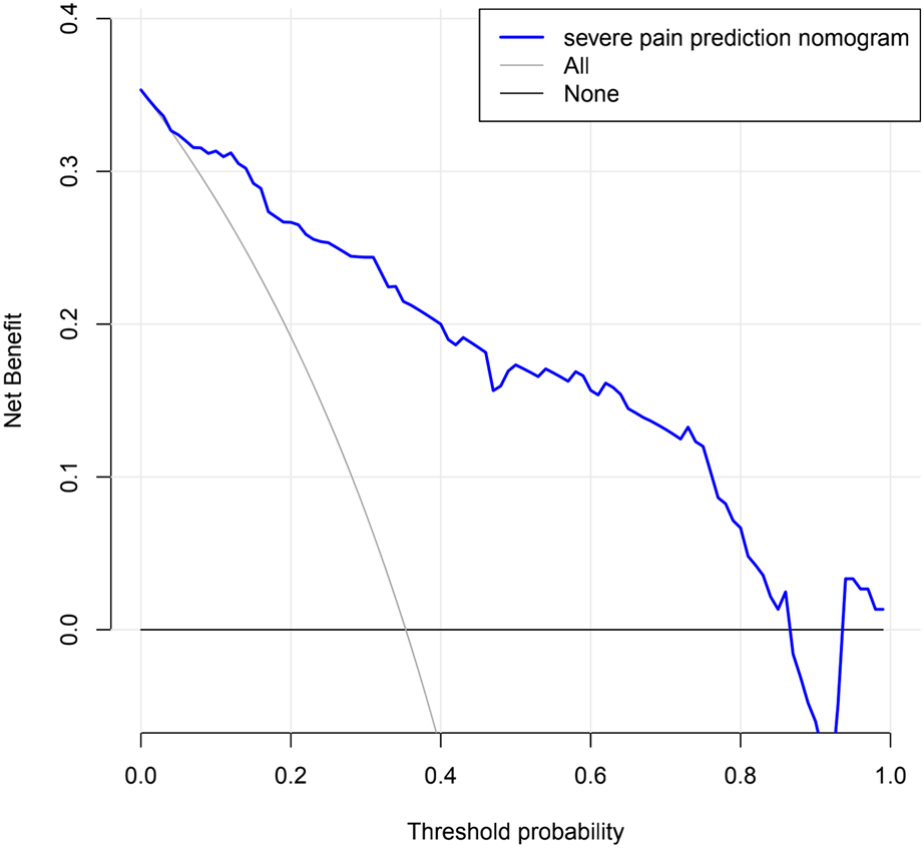
DCA curve of nomogram of modeling group.

Decision curve analysis of a nomogram predicting the risk of severe pain in KOA patients. The decision curves showed that the nomogram could effectively predict the risk of developing severe pain in KOA patients if the threshold probabilities for patients and physicians were >0.1 and <0.86, respectively. In this range, it showed excellent clinical effect based on the predicted risk of developing severe pain in KOA patients. The details are presented in Figure 6.

## Discussion

Due to the increasing prevalence of global obesity and the growing aging population, the number of KOA patients has increased tremendously worldwide. Pain is one of the most important clinical manifestations in patients with KOA and is often a key factor influencing surgical treatment of TKA patients. The mechanisms of pain associated with knee OA are complex, and local structural changes in the knee, such as synovitis, degree of bone marrow pathology, and deformity changes in the kneecap, are postulated to cause or induce different kinds of pain (37).

To our knowledge, this is the first study to develop and validate a risk probability model for predicting the occurrence of severe pain in KOA patients. In both the training and validation cohorts, the nomogram showed satisfactory agreement, and DCA revealed that it had good clinical applicability. Nomograms are widely used for various diseases, primarily because of their ability to reduce statistical predictive models to a single numerical representation of the probability of an event (e.g., diagnosis or recurrence) tailored to an individual patient′s profile. The user-friendly and easy-to-understand graphical interface used to generate these estimates allows the use of nomograms in clinical practice to inform clinical decision making (38). In our study, KOA patients with a high risk of severe pain should be treated early to delay suffering from severe pain or eventual TKA surgery. This will reduce social and personal financial stress. Nomogram provide important biologically and clinically integrated models that allow implementation of personalized medicine in clinical practice (39). Currently, there are no effective models for predicting the occurrence of severe pain in patients with KOA, and the risk factors influencing pain in KOA are not completely known. For these reasons, we aimed to develop and validate a nomogram for predicting the probability of occurrence of severe pain for KOA patients with high efficiency, high accuracy, and as many variables as possible to demonstrate the predictive power of the nomogram.

Research has revealed a strong link between BMI and KOA. It has been shown that obesity can increase the occurrence of KOA and the progression of pain (40, 41). High BMI can interfere with or can modify the occurrence of pain in KOA patients (42). Several studies have demonstrated that BMI is a predictor of KOA pain (43). This is consistent with our findings. This study demonstrated the importance of BMI in predicting the occurrence of severe pain in KOA patients, with KOA patients with high BMI having a considerably higher risk of severe pain than those with low to moderate BMI. In addition, many studies have reported that bone marrow lesions (BMLs) are closely associated with pain in KOA (10, 44–46). These findings are consistent to the present results. Our study also found that the higher the BML score, the more severe the lesion, and the greater the probability of severe pain in KOA patients. Some studies have shown that biomechanical factors such as meniscal displacement may trigger the development of KOA and accelerate the progression of the patient’s pain level (47). The nomogram has long been used to predict clinical disease. The nomogram is an easy-to-use and accurate prediction tool that has been widely used in medical research, particularly for the diagnosis or prognosis of various diseases, primarily because the nomogram contains many correlates that allow the statistical prediction model to be reduced to individual scores that can be customized for individual patients, with the final score estimating the probability of an endpoint (such as diagnosis or recurrence). Nomogram′s graphical interface is easy to understand and easy to use when communicating treatment options or prognostic strategies to patients. It thus provides clinicians with more optional information for decision-making (38). The nomogram fulfills the need for an integrated biological and therapeutic model, as well as the need for personalized treatment that can provide unique patient prognostic predictions (39). Validation of the nomogram before a clinical application is essential for physicians (48). As a result, nomograms have become a reliable tool for clinical decision-making and predicting outcomes in many diseases.

Our study found that KOA patients with a longer duration of disease, meniscal damage or higher meniscal scores, high synovitis scores, and right-sided KOA had a higher probability of severe pain. In addition, the more influential the bone wear, the greater the relative likelihood of obtaining severe pain. In the constructed nomogram we developed, the severity of bone wear was one of the most important factors influencing the occurrence of severe pain in KOA patients, followed by the synovitis score and whether the meniscus was displaced. These factors can be used to predict the risk of severe pain in KOA patients.

There are some limitations to our study. First, this was a retrospective study, and therefore, there is some inherent bias. Moreover, many factors such as MRI and x-ray imaging data, were not available. Therefore, further studies are needed to validate our findings through multicenter internal validation.

## Conclusion

In this study, higher BMI, synovitis score, BML score, bone wear score, meniscus score, in addition to right-sided KOA or presence of meniscal displacement and longer duration of KOA were found to be risk factors for the development of severe pain in patients with KOA. A nomogram comprising eight predictors showed good ability to predict the risk of severe pain in patients with KOA more accurately.

## Data Availability

The raw data supporting the conclusions of this article will be made available by the authors, without undue reservation.
