# Corrigendum: A nomogram based on radiological features of MRI for predicting the risk of severe pain in patients with osteoarthritis of the knee

**DOI:** 10.3389/fsurg.2025.1571517

**Published:** 2025-03-14

**Authors:** Zhuce Shao, Zhipeng Liang, Peng Hu, Shuxiong Bi

**Affiliations:** Department of Bone and Joint, Third Hospital of Shanxi Medical University, Shanxi Bethune Hospital, Shanxi Academy of Medical Sciences, Tongji Shanxi Hospital, Taiyuan, China

**Keywords:** KOA, K–L, nomogram, forecast, pain, TKA

A Corrigendum on A nomogram based on radiological features of MRI for predicting the risk of severe pain in patients with osteoarthritis of the knee By Shao Z, Liang Z, Hu P and Bi S (2023). Front. Surg. 10:1030164. doi: 10.3389/fsurg.2023.1030164

**Error in Figure/Table**

In the published article, there was an error in Table 2 as published. One of the numbers was incorrect when inserted in the table, the number marked in red in the table below (in the original text it is the *p*-value corresponding to the time in Table 2), was written incorrectly as 3.261, the real correct number is 0.030. This error doesn't affect all the tables and pictures that are expressed and carried out later on, nor does it affect any of the analysis and results and conclusions of this study. The corrected Table 2 and its caption “Logistics regression univariate analysis” appear below.

**Table 2:** Logistics regression univariate analysis.

Variable	Univariate analysis	OR	95% CI	*P* value
	*β*			
BMI	0.515	1.674	0.876–3.5041	0.152
Laterality	0.501	1.650	0.843–3.260	0.145
Time	0.766	2.151	1.081–4.312	0.030
Meniscus_score	0.507	1.661	0.796–3.612	0.186
BML	1.66	5.257	2.4920–11.464	0.000
Synovitis_score	1.141	3.133	1.566–6.375	0.001
Bone_wearscore	2.393	10.951	4.736–27.496	0.000
Meniscus_displacement	2.065	7.884	3.778–17.189	0.000

The authors apologize for this error and state that this does not change the scientific conclusions of the article in any way. The original article has been updated.

**Text Correction**

In the published article, there was a clerical error, whereby the description was not precise enough.

A correction has been made to **Results,** “Logistics univariate and multivariate regression analysis of severe pain in patients with KOA”. This sentence previously stated:

“Logistic univariate regression showed that BML score, synovitis score, bone wear score, and meniscus displacement were more predictive and were independent risk factors for severe pain in patients with KOA.”

The corrected sentence appears below:

“Logistic regression results showed that factors including BML score, synovitis score, bone wear score and meniscus displacement among others were more predictive of severe pain and were risk factors for severe pain in patients with KOA.”

The authors apologize for this error and state that this does not change the scientific conclusions of the article in any way. The original article has been updated.

